# Glucocorticoids and cell fate in the developing brain: Neuroendocrine mechanisms shaping developmental trajectories

**DOI:** 10.1111/jne.70142

**Published:** 2026-02-06

**Authors:** Helen Eachus

**Affiliations:** ^1^ Institute of Health and Neurodevelopment, College of Health & Life Sciences, Aston University Birmingham UK

**Keywords:** brain development, cell fate, glucocorticoids, neurogenesis, stress

## Abstract

Early life stress (ELS) is a major risk factor for later psychiatric and neurological disorders. Glucocorticoids (GCs), the hormonal end‐products of the neuroendocrine stress response, are central mediators of this risk, influencing how the developing brain grows and adapts. Research has shown that GCs affect processes such as cell proliferation, neuronal survival, and maturation, but much less attention has been given to whether they also shape *cell fate*—the developmental choices that determine whether stem and progenitor cells give rise to neurons, astrocytes, oligodendrocytes, or other specialised lineages. In this perspective, I argue that cell fate provides a valuable new lens for understanding how stress becomes embedded in brain architecture. Because GCs act directly on neural stem and progenitor populations, it is plausible that their influence extends beyond the quantity of cells produced, to the identities that emerge. I outline an initial framework for interpreting potential effects of GCs on fate, review emerging evidence from different model systems, and consider mechanisms by which stress hormones could alter developmental trajectories. By focusing on fate, this article highlights a novel dimension of neuroendocrine influence on brain development, with implications for how early experiences confer vulnerability, or resilience, to later mental health outcomes.

## 1 INTRODUCTION

Early life stress (ELS) is one of the most potent environmental risk factors for long‐term psychiatric and neurological vulnerability.^1^, ^2^, ^3^ A wealth of evidence links ELS with wide‐ranging alterations in brain development and function, affecting both structural maturation and the organisation of stress‐responsive circuits.^1^, ^4^, ^5^, ^6^ At the centre of this biology lies the Hypothalamo–Pituitary–Adrenal (HPA) axis, whose end‐product glucocorticoids (GCs) exert widespread actions on the developing brain.^7^, ^8^ GCs act through glucocorticoid and mineralocorticoid receptors (GR and MR) to regulate diverse neurodevelopmental processes, acting on transcriptional, epigenetic, and signalling pathways with the capacity to shape emerging neural architecture.^9^, ^10^, ^11^

The developing brain is particularly sensitive to GC exposure and foundational work in rodents and humans has demonstrated that elevated GCs can reshape brain development at multiple levels.^12^, ^13^, ^14^, ^15^ Early rodent studies reported that excess GCs reduce hippocampal neurogenesis,^16^ alter dendritic and spine architecture,^17^ and disrupt the maturation of limbic and cortical circuits.^18^, ^19^ Meanwhile ELS paradigms revealed impairments in cell proliferation,^20^ migration,^21^ and synaptic plasticity,^22^ particularly in hippocampal and prefrontal regions. In humans, antenatal synthetic GC treatment and exposure to maternal stress have been associated with altered cortical thickness,^23^ altered amygdalar and hippocampal morphology,^24^, ^25^ and long‐term cognitive and emotional differences.^26^, ^27^ GC exposure in early life is known to affect multiple phases of brain development and maturation, including synaptic pruning^17^ and myelination.^28^ Importantly, studies have linked GC‐induced alterations in neurogenesis with broader phenotypes,^29^, ^30^ including changes in circuit architecture and behavioural responsiveness, implicating neurogenesis as a potential mediator of diverse effects of GCs on the brain.^31^, ^32^, ^33^

The impact of GCs on neurogenesis is also strongly dependent on developmental timing. During the prenatal period, when neurogenesis is at its peak, elevated GC or ELS exposure has been shown to reduce progenitor proliferation,^34^ impair neuronal differentiation,^35^ and shift the balance between neuronal and glial output^36^ in multiple rodent models. Postnatally, when neurogenesis continues in regions such as the hippocampus and hypothalamus, heightened GC levels and stress can suppress neuronal production,^37^ influence progenitor pool maintenance,^38^ and modify the maturation trajectories of emerging cell populations.^39^ Adolescence represents another sensitive window in which GC fluctuations can dampen hippocampal neurogenesis, impair neuroplasticity, and alter cognitive function and behaviour.^13^ Findings from in vitro systems similarly show that the stage at which progenitors encounter GCs profoundly shapes their neurogenic responses,^40^ underscoring the importance of temporal context in interpreting GC effects on neurogenesis.

In addition to these temporal effects, GC effects are spatially heterogeneous. Although the hippocampus has traditionally dominated studies of GC effects on neurogenesis, largely because it expresses high levels of GRs in rodents,^41^ emerging work indicates that other regions may be equally or more sensitive depending on developmental stage and species. For example, primate data revealed high MR but relatively low GR expression in the hippocampus, with higher GR levels in the hypothalamus, prefrontal and entorhinal cortices, and cerebellar cortex,^42^ suggesting that the hippocampus may not be the most GC‐sensitive brain region in primates. While studies investigating the effects of GC exposure across brain regions are rare, our recent zebrafish study showed that early GC exposure disproportionately affected hypothalamic progenitor dynamics,^43^ and in rodents, ELS was demonstrated to have divergent transcriptional effects on neurogenesis pathways across different reward circuits.^44^ Rodent MRI studies highlighted the vulnerability of the PFC to ELS‐induced shrinkage, above other regions,^45^ meanwhile, prenatal GC exposure in rats led to increased volume of the bed nucleus of the stria terminalis (BNST) but a reduction in the amygdala.^46^ Such studies highlight that GCs may drive distinct consequences for neuronal development of key brain regions implicated in stress regulation. Such spatiotemporal specificity provides important context for interpreting GC‐induced developmental outcomes.

These decades of research have established that stress and GCs can alter neurogenesis, gliogenesis, and overall brain plasticity, with downstream consequences for circuit formation and behaviour.^12^, ^13^, ^14^, ^15^ Most work to date has focused on how GCs modulate proliferation, survival, or maturation of neural cells.^8^, ^32^, ^47^ However, these findings also reveal a deeper challenge: GC effects are highly dependent on developmental stage, cell type, and brain region. Such diversity in GC responses has shaped the field's focus towards understanding *how much* neurogenesis occurs under stress, or how neuronal and glial populations grow, shrink, or mature differently. However, this emphasis has meant that other important dimensions of progenitor behaviour, such as how GCs influence the *types* of cells that are generated, have received far less attention. Historically, lineage decisions have been difficult to monitor in vivo, and early GC studies naturally centred on more readily measurable outcomes such as proliferation rates or dendritic structure. As a result, potential GC effects on cellular identity have remained largely unexamined.

Cell fate represents a mechanistically distinct and conceptually important aspect of neurodevelopment. Whereas changes in proliferation or survival might alter the *number* of cells produced, fate decisions determine the *identities* of those cells and therefore the composition of emerging neural circuits. While external stimuli may drive shifts in abundance of specific cell populations in the brain, such alterations may arise from changes in proliferation or cell death without altering the identities produced, whereas fate changes would imply a fundamental reprogramming of progenitor output. If GCs bias or redirect lineage output, the resulting shifts in neuronal or glial subtypes could influence connectivity, excitability, or circuit function in ways that cannot be inferred from cell number alone. Recognising cell fate as a separate aspect of GC sensitivity therefore provides a valuable lens through which to interpret developmental outcomes that may have previously been attributed solely to changes in neurogenesis or maturation.

Emerging evidence is beginning to suggest that experiences including exposure to ELS or GCs may indeed bias or redirect fate decisions, providing a new perspective on how stress becomes embedded in neural architecture. The effects of ELS and GCs on the brain are known to be cell type‐specific.^48^ A key target of GCs is neural stem and progenitor cells (NSPCs),^49^ the very populations where lineage choices are made; thus, it is plausible that stress hormones could shape not only the pace of neurogenesis and gliogenesis but also the repertoire of identities that ultimately emerge. While direct evidence remains limited, early findings hint that GCs may bias developmental trajectories in ways that alter the cellular composition of the brain, with potential for lasting consequences.

Here I discuss an outline for understanding how stress and GCs influence cell fate in the brain. I distinguish direct cell fate reprogramming from subtler biases and within‐lineage shifts in neurogenesis and synthesise some of the recent evidence across model systems. I explore some key molecular mechanisms that underpin these effects and outline future approaches for dissecting how transient hormonal cues might translate into enduring developmental trajectories. By focusing on fate, I aim to highlight a novel lens through which to understand how stress hormones sculpt brain architecture and confer vulnerability to psychiatric disorders.

## 2 CELL FATE IN THE BRAIN

The influence of stress and GCs on neurogenesis has been widely demonstrated, with numerous studies reporting alterations in brain structure, cytoarchitecture and the relative abundance of different cell populations.^31^, ^47^, ^50^ These observations raise the question of the underlying mechanisms: do such shifts reflect altered dynamics of proliferation, differentiation or survival within predetermined lineages, or in some cases could they reflect changes in the fundamental fate decisions of developing cells? Distinguishing between these possibilities is essential for understanding how ELS leaves enduring imprints on brain development, yet in many cases the underlying processes remain unresolved.

At its core, cell fate refers to the stable identity that a progenitor or precursor ultimately adopts, whether as a neuron, astrocyte, oligodendrocyte, or a more finely defined subtype.^51^ Under normal conditions, these identities emerge through coordinated transcriptional programmes, epigenetic regulation, extrinsic cues and temporally ordered patterns of neurogenesis and gliogenesis.^51^ Direct changes to cell fate can be defined as alterations that redirect the developmental trajectory of progenitor or precursor cells towards an identity they would not otherwise have adopted, or that fundamentally reshape the balance of outcomes available to them. Such mechanisms imply a primary reprogramming of cell identity itself, rather than secondary modulation of proliferation, maturation, or survival within already specified lineages.

**FIGURE 1:**
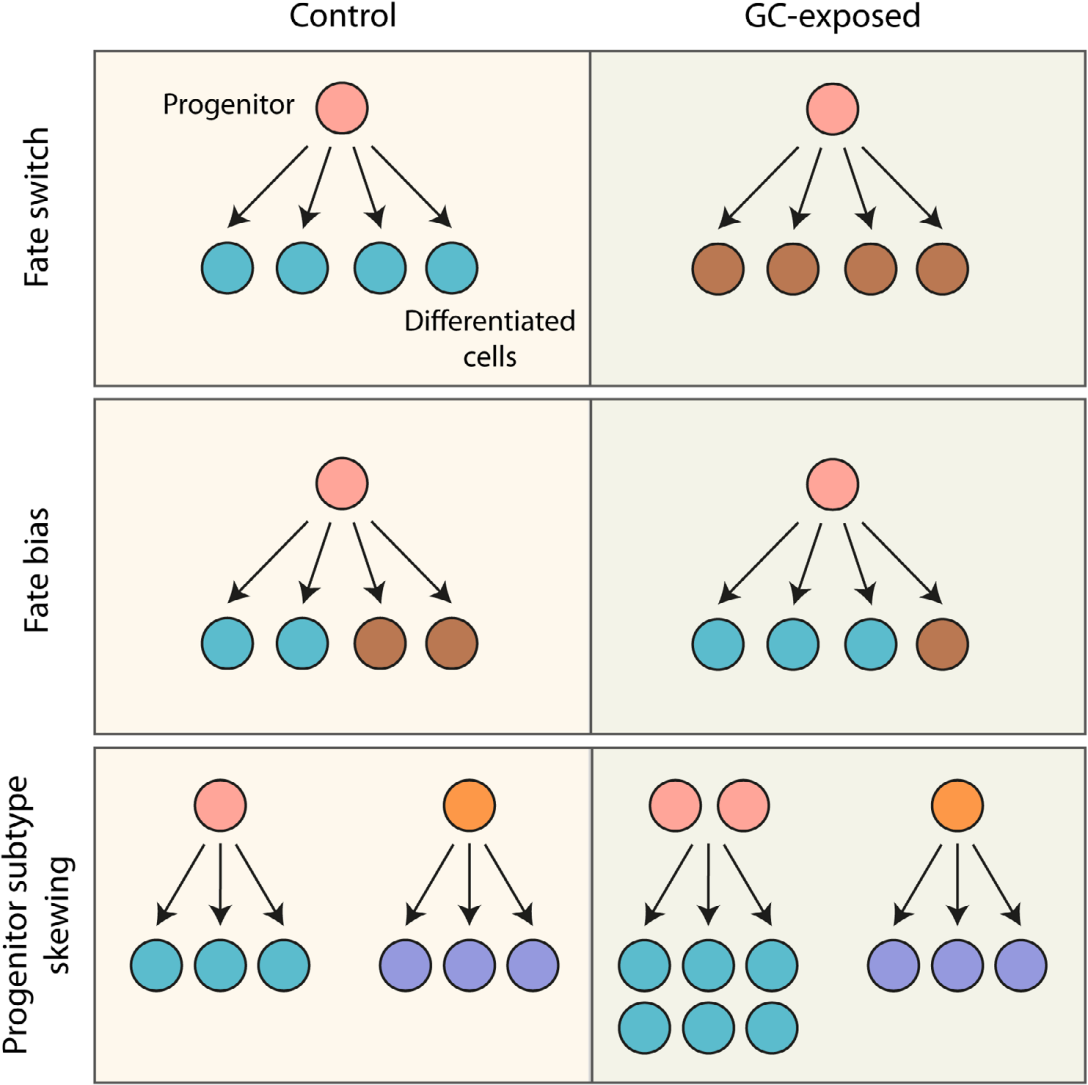
Conceptual framework for GC‐mediated modulation of cell fate in the brain. A fate switch may occur whereby a progenitor cell produces a differentiated cell type that it would not normally be primed to produce. A fate bias may occur when a multipotent progenitor cell adopts one of its possible fates more frequently than it normally would. Progenitor subtype skewing may occur where a bias occurs at the level of the progenitor subtype. Here a pool of fate‐restricted progenitors is amplified, expanding the output of that lineage.

The most decisive form of direct fate change is a *fate switch* (Figure 1), in which a progenitor that would ordinarily follow one developmental trajectory is actively redirected to adopt a qualitatively different identity.^52^ For example, a neural precursor that would typically generate a neuron is instead differentiating into a glial cell. Such reprogramming requires intervention at the level of fundamental transcriptional and signalling networks and has been demonstrated experimentally, for instance by in vivo electrical stimulation that forces mesenchymal stem cells to adopt a neural fate.^53^ While this striking demonstration illustrates that true fate switching is possible, it is unlikely to occur under physiological conditions in the developing brain.

More commonly, developing cells do not undergo such wholesale reprogramming, but might instead show *fate biasing* (Figure 1). In this scenario, multipotent progenitors retain the capacity to generate multiple lineages, yet the balance of outcomes is shifted,^54^ such that a progenitor pool may preferentially produce inhibitory rather than excitatory neurons, for example. Here, no individual progenitor loses or gains potential, but rather the distribution of fates across the population is subtly redirected. Another possibility is *progenitor subtype skewing* (Figure 1), in which the relative abundance of progenitor subtypes is altered without changing their intrinsic programmes.^55^ For example, if stress or GC exposure preferentially expands progenitors already restricted to a neurogenic trajectory, the overall lineage output will appear fate‐biased, even though individual progenitors continue to generate their expected cell types.

By contrast, not all changes in cell population balance necessarily reflect true alterations in fate specification.^56^ In many cases, stress and GC exposure may act more indirectly, for example by altering the relative rates of proliferation, survival, or maturation within pre‐established lineages. Such mechanisms can still shift the abundance of particular cell types, but without fundamentally redirecting progenitor identity. Temporal disruptions to fate patterning provide a useful illustration: progenitors in the cortex normally follow a sequential programme, generating early‐born deep‐layer neurons before later switching to upper‐layer neurons and eventually glia.^57^ If this transition occurs prematurely or is delayed, the resulting output of cell types may be markedly reweighted, even though each fate still lies within the progenitor's intrinsic repertoire. Similarly, accelerated maturation of one cell type could yield an apparent enrichment of mature cells, yet without any underlying change in lineage choice. These more indirect routes exemplify that population‐level alterations can emerge through diverse mechanisms, only some of which reflect a reprogramming of fate.

## 3 GLUCOCORTICOID‐LINKED MODULATION OF CELL FATE

Evidence for GC‐induced fate bias is provided by in vitro, organoid and in vivo studies (summarised in Table 1), with strong initial support found in earlier work in simple cellular systems. Jameson et al.^58^ demonstrated that exposure to dexamethasone, a synthetic glucocorticoid, alters neurotransmitter phenotype specification in PC12 cells, a rat adrenal pheochromocytoma line commonly used as a neuronal differentiation model. Under standard conditions, these cells can differentiate towards either a cholinergic or a catecholaminergic fate, reflecting a developmental decision point that parallels aspects of sympathetic lineage diversification. Strikingly, chronic GC treatment biased this trajectory away from the cholinergic identity and towards an adrenergic phenotype, as indicated by reduced choline acetyltransferase levels and enhanced tyrosine hydroxylase levels. Although PC12 cells represent a reductionist model, the findings are important in showing that GCs can actively reshape the balance between alternative neuronal identities.

**TABLE 1:** Summary of model systems, glucocorticoid manipulations, developmental windows and key cell‐fate outcomes discussed in this review.

Model system	GC manipulation	Developmental window	Cell‐fate classification	Fate‐related outcome	Possible mechanism	References
PC12 neuronal model	Dexamethasone	In vitro differentiation	Fate bias	Shift from cholinergic → catecholaminergic identity	↓ChAT; ↑TH	Jameson 2006^58^
Human hippocampal progenitor line	Cortisol (MR vs. GR activation)	In vitro differentiation	Fate bias	MR: ↑astrocytes, ↓neurons; GR: ↓neurons	MR → Notch/Hes; GR → suppresses TGFβ–SMAD2/3 and Hedgehog	Anacker 2013^59^
Human neural organoids	Dexamethasone	Early neurodevelopment	Fate bias	↑Inhibitory neurons, ↓excitatory neurons	*PBX3* mediates GC‐responsive lineage bias	Dony 2025^61^
Adult rat dentate gyrus	Restraint stress and corticosterone	Adult neurogenesis	Fate bias	↑Oligodendrocytes; ↓neurons	GR‐dependent oligodendrogenic programme	Chetty 2014^65^
Adult mouse hippocampus	Social isolation (↑GCs) vs. enrichment (↓GCs)	Adult neurogenesis	Fate bias	Isolation: ↑astrocytic differentiation and ↑RGL pool; Enrichment: ↑neurons, ↓astrocytic differentiation and ↓RGL pool	Experience‐linked modulation of NSC allocation	Dranovsky 2011^66^
Zebrafish hypothalamus	Optogenetic elevation of endogenous GCs	Early development (5–13 dpf)	Fate bias within neuronal subtypes and altered neurogenesis dynamics	Fate: 5 dpf: ↑*npy*, ↑*th*, ↓*crh*; 13 dpf: ↑*pmch*; ↓*agrp*, ↓*cart* neurons. Neurogenesis dynamics: precocious proliferation followed by failed differentiation	Altered *rx3* and developmental transcriptional programme	Eachus 2024^43^
Human cerebral organoids and mouse cortex	Dexamethasone	Early corticogenesis	Progenitor subtype skewing	↑PAX6+/EOMES+ basal progenitors → ↑upper‐layer neurons	ZBTB16 upregulated by GC	Krontira 2024^70^
Mouse hypothalamus (PVN)	Limited bedding/nesting (ELS)	Early development	Altered CRF neuron subtype balance	↓GABAergic neuron cluster, ↑ glutamatergic neuron cluster	Altered transcriptional programmes	Short 2023^71^
Human vmPFC post‐mortem	Childhood maltreatment	Long‐term developmental imprinting	Within‐lineage bias/maturation dynamics	↑Mature oligodendrocytes; ↓immature states	ELS‐linked acceleration of oligodendrocyte maturation	Tanti 2018^72^

Anacker and colleagues^59^ provided compelling receptor‐ and dose‐dependent data supporting that GCs can directly bias fate decisions in human hippocampal progenitors. Using an immortalised human hippocampal progenitor cell line, they demonstrated that cortisol exerts receptor‐specific and dose‐dependent effects on lineage outcomes. At low concentrations, where MR activity dominates, cortisol promoted progenitor proliferation while reducing neuronal differentiation and increasing astrocytic output. This effect was abolished by MR antagonism and reproduced by an MR agonist, indicating that MR activation actively drives a bias towards gliogenesis. In contrast, at higher concentrations where GR activity prevails, cortisol reduced both proliferation and neuronal differentiation, but without a compensatory increase in astrocytic fate. These findings highlight a divergence between MR‐ and GR‐mediated signalling, with MR activation exerting a true fate bias towards glial lineages, whereas GR activation primarily suppresses neuronal output.

Mechanistically, the study linked these effects to specific signalling cascades. MR‐mediated gliogenic bias was associated with activation of Notch/Hes signalling, consistent with the well‐established role of this pathway in promoting glial differentiation.^60^ GR activation, by contrast, suppressed TGFβ‐SMAD2/3 signalling, a pathway normally required for neurogenesis. Both receptor pathways converged on inhibition of Hedgehog signalling, and pharmacological activation of Hedgehog rescued neuronal differentiation, implicating this pathway as a critical mediator of GC‐induced fate changes. Importantly, similar reductions in Hedgehog and TGFβ‐SMAD2/3 activity were observed in the hippocampi of prenatally stressed rats, underscoring the translational relevance of the findings. Taken together, this study provides compelling evidence that cortisol can actively re‐route lineage decisions in human hippocampal progenitors, with MR promoting a shift from neuronal to astrocytic fate and GR constraining neuronal production through suppression of pro‐neurogenic signalling.

Complementary work in organoid models, such as the recent study by Dony et al.,^61^ provides one of the clearest demonstrations to date that chronic GC exposure can bias neuronal fate decisions in human brain development. Using unguided and ventralised human neural organoid models, they show that prolonged GC treatment drives a shift in differentiation from excitatory towards inhibitory neurons. This effect was not a general developmental arrest but rather a redistribution of lineage output, with excitatory neuron populations reduced and inhibitory lineages disproportionately expanded. The ventralised organoid context was especially informative, as it recapitulates environments where inhibitory trajectories are normally favoured,^62^ and here the GC‐induced skew was most pronounced, reinforcing the idea that stress hormones act to amplify pre‐existing lineage biases. Importantly, these fate changes were consistently observed across organoid replicates, providing robust evidence that GC exposure actively remodels excitatory–inhibitory balance during early human neurodevelopment.

To dissect underlying mechanisms, the authors integrated single‐cell transcriptomic and epigenomic profiling with in silico perturbation of gene regulatory networks. This highlighted *PBX3* (*Pre‐B‐cell leukaemia transcription factor 3*) as a central node responsive to GC exposure. Although *PBX3* is most strongly expressed in neurons rather than progenitors, perturbation analyses suggest that changes in PBX3 activity can both destabilise progenitor identity and selectively enhance inhibitory lineage differentiation. This raises the possibility that PBX3 functions as a mediator of stress‐induced fate bias, not by initiating the change at the progenitor stage per se, but by amplifying lineage trajectories as cells exit the progenitor pool. In this way, the work by Dony et al. highlights a mechanistically grounded example of a cell fate bias alteration, whereby GCs skew the normal repertoire of neuronal fates produced without entirely redirecting progenitor potential. Given the link between neurodevelopmental disorders and altered excitation‐inhibition balance in the brain,^63^, ^64^ GC‐induced neuronal fate bias presents a potential mechanism underlying GC‐linked neurodevelopmental disorders.

The above‐mentioned in vitro and organoid studies are also supported by in vivo evidence. Chetty et al.^65^ provide strong evidence that stress and GCs can bias adult hippocampal progenitor fate towards oligodendrogenesis at the expense of neurogenesis. Using a one‐week paradigm of repeated restraint stress in adult rats, combined with BrdU lineage tracing, they demonstrated a significant increase in BrdU+ oligodendrocytes and a concomitant reduction in BrdU+ neurons within the dentate gyrus. This finding was reproduced by systemic corticosterone administration, directly implicating stress hormones as the mediators of this lineage shift. Importantly, they validated these results using Nestin‐CreER/YFP genetic fate mapping, showing that Nestin‐expressing adult neural stem cells themselves were redirected towards an oligodendroglial fate under corticosterone exposure, ruling out an indirect effect driven by non‐stem cell populations. Complementary in vitro experiments using cultured adult hippocampal NSCs revealed the same bias: corticosterone promoted oligodendrocyte differentiation and suppressed neuronal differentiation. Mechanistically, these effects were shown to be GR dependent, as blocking GR signalling abolished the fate bias. Interestingly, corticosterone exposure also modulated the transcriptional landscape of NSCs, altering expression of pro‐oligodendrogenic factors while leaving neurogenic factors largely unaffected, suggesting a selective transcriptional programme through which GR signalling enhances oligodendrogenesis. Taken together, these findings demonstrate that elevated GC signalling can act directly on adult hippocampal progenitors to reallocate lineage potential away from neuronal differentiation and towards oligodendrocyte production, providing a clear mechanistic example of GC‐induced fate bias in the adult brain.

Dranovsky and colleagues^66^ reported a detailed investigation into how environmental experience can remodel the balance of neural stem cell fates in the adult hippocampus. Using indelible Nestin‐CreER fate mapping in mice to label radial glia–like stem cells (RGLs), they examined the impact of social isolation, a robust psychological stressor that is known to elevate GC levels,^67^ versus environmental enrichment, which typically reduces GC activity.^68^ In animals isolated for 1 month, they observed a surprising expansion of the GFAP^+^ RGL pool alongside an increase in the proportion of GFAP^+^ stellate astrocytes, without a corresponding rise in newborn neurons. This pattern suggests that isolation promotes self‐renewal or astrocytic differentiation at the expense of neurogenesis. Conversely, environmental enrichment shifted fate decisions towards increased neuronal output, with a reduced proportion of RGL and astrocytes, an apparent fate trade‐off where increased neurogenesis was balanced by decreased glial and stem‐cell output.

Although the study did not directly manipulate GC signalling, the bidirectional effects of isolation and enrichment are consistent with GC‐dependent modulation of lineage trajectories. This is supported by other work linking elevated GC levels to reduced neurogenesis and enhanced gliogenesis,^59^ and conversely by evidence that lowered GC levels promote neuronal differentiation.^69^ In this light, the finding that isolation expanded the RGL pool and increased astrocytic differentiation, whereas enrichment promoted neuronal output at the expense of stem cells and glia, aligns with the idea that experience‐dependent modulation of the HPA axis indirectly biases hippocampal fate decisions. Importantly, these effects represent genuine alterations in fate allocation rather than simple changes in proliferation or survival, underscoring that adult hippocampal stem cells adjust not only the number but also the identity of their progeny in response to environmental cues.

Building on these mammalian and in vitro examples, our recent work in zebrafish has demonstrated that elevated GC during early development can specifically redirect hypothalamic neuronal fate.^43^ Using an optogenetic approach to chronically stimulate endogenous GC production, our work revealed that the hypothalamus is especially sensitive to GC exposure. At 5 dpf (days post fertilisation), GC‐exposed larvae showed a striking shift in lineage allocation, with increased numbers of *npy*‐ and *th*‐expressing neurons, accompanied by a reduction in *crh*‐expressing hypothalamic neurons. This finding indicates that GC can bias differentiating progenitors away from stress axis output neurons and towards alternative peptidergic and catecholaminergic fates.

By 13 dpf, the pattern of fate bias had evolved further, with an overproduction of *pmch* neurons and concurrent reductions in *agrp*‐ and *cart*‐expressing hypothalamic populations. These temporally distinct effects suggest that GC does not simply alter hypothalamic maturation but rather produces sustained lineage biases that reshape circuit composition across developmental windows. Supporting this, transcriptional analyses indicated altered regulation of developmental factors such as *rx3*, pointing to a mechanism whereby GC may directly modulate progenitor trajectories. Together, these data provide evidence that early life GC exposure can induce fate biasing within the hypothalamus, reinforcing the view that GC acts as a powerful developmental signal with lasting consequences for neuroendocrine identity.

Taken together, these studies converge on the principle that GCs do not merely suppress neurogenesis or alter overall cell numbers but can also redirect lineage allocation in diverse neural contexts. From human organoids to rodent hippocampus, immortalised progenitor lines, and zebrafish hypothalamus, there is a recurring pattern of cell *fate bias*. Although the specific outcomes depend on developmental stage, brain region, and receptor signalling context, the unifying insight is that stress hormones may act as developmental signals with the capacity to reroute cellular trajectories. This framework places fate bias at the centre of how GCs may sculpt neural circuits, with potential long‐term consequences for brain function and vulnerability to stress‐related disorders.

## 4 GLUCOCORTICOID‐MEDIATED ALTERATION OF NEUROGENESIS DYNAMICS

Beyond overt fate switching and bias, GCs can reshape lineage outcomes indirectly, by skewing progenitor subtype composition, altering proliferation dynamics, or tuning transcriptional programmes. These mechanisms may not produce clear trade‐offs between identities, but rather in more subtle shifts in progenitor dynamics or lineage balance that cumulatively alter the cellular composition of brain regions.

Krontira et al.^70^ exemplify this. Using human cerebral organoids and complementary mouse in vivo assays, they show that chronic GC exposure selectively expands a basal progenitor subtype co‐expressing PAX6 and EOMES, a population linked to upper‐layer neurogenesis, without detectable changes in other progenitor pools. This progenitor skew associates with increased production of upper‐layer cortical neurons. Mechanistically, they show that GCs upregulate ZBTB16, and ZBTB16 overexpression recapitulates the expansion of PAX6^+^/EOMES^+^ progenitors; meanwhile reducing ZBTB16 blunts the effect. Notably, the study does not directly lineage‐trace the expanded progenitors to the increased upper‐layer neurons, and compensatory adjustments among other progenitor subtypes may arise in vivo. However, the data suggest a GC‐driven progenitor subtype skewing and proliferative bias mediated by ZBTB16, potentially driving an indirect route to altered neuronal output.

A further demonstration of GC‐linked modulation of neurogenesis dynamics emerges from our recent work in zebrafish, which revealed that early GC exposure alters not only hypothalamic fate allocation (as described above) but also the temporal progression of neurogenesis itself.^43^ Chronic elevation of endogenous GCs induced an early surge in hypothalamic progenitor proliferation, producing a transient expansion of the stem/progenitor pool. However, this precocious proliferative phase was followed by impaired differentiation and a subsequent decline in proliferative capacity, suggesting that sustained GC signalling disrupts the normal transition from progenitor expansion to neuronal maturation. These temporally dissociated effects, initial acceleration of progenitor activity followed by failed differentiation, illustrate how GCs can perturb the dynamics of neurogenesis potentially independently of, and in parallel to, changes in fate bias. Such findings highlight that GC exposure can reshape lineage trajectories not only by influencing identity choices but also by distorting the developmental pacing and maturation states of progenitor populations.

Building on this theme of selective modulation, Short and colleagues^71^ used single‐cell RNA sequencing of the hypothalamic paraventricular nucleus (PVN) to examine how early life adversity reshapes corticotropin‐releasing factor (CRF)‐expressing neurons. They found that adversity biased the balance of 5 distinct CRF neuronal subtypes, with a reduction in cluster 2 GABAergic neurons and a corresponding increase in cluster 4 glutamatergic neurons. This latter cluster was distinct from the other glutamatergic CRF populations in lacking vasopressin co‐expression, exhibiting unique growth‐associated transcriptional signatures, and showing a dispersed rather than clustered spatial distribution. Interestingly, adversity‐induced transcriptional changes were largely confined to cluster 1 glutamatergic CRF neurons, suggesting highly selective vulnerability within the PVN. While CRF neuronal subtypes appear to differ in their transcriptional programmes, morphologies, and spatial organisation within the PVN,^71^, ^72^ their developmental origins remain unresolved. Thus, while Short et al. demonstrate that ELS may reshape subtype balance, it is not yet clear whether this reflects altered lineage allocation, differential survival, or activity‐dependent remodelling. Collectively, the findings highlight a subtle form of GC‐linked fate modulation that operates within a defined neuroendocrine lineage, expanding our understanding of how stress sculpts hypothalamic circuits.

Complementary evidence comes from human post‐mortem studies, which provide insight into how severe ELS may leave lasting imprints on glial lineage dynamics. Tanti et al.^73^ examined the ventromedial prefrontal cortex of individuals with a history of childhood abuse and reported a shift within the oligodendrocyte lineage. Specifically, they observed increased numbers of mature oligodendrocytes accompanied by a reduction in immature cells, while progenitor numbers were unchanged. The authors proposed that progenitors may undergo compensatory proliferation to sustain the pool despite accelerated differentiation, although no direct evidence supports this. These findings align with the idea of a within‐lineage bias, whereby ELS skews the balance of maturation states within a single lineage. Given the well‐established links between childhood maltreatment, HPA axis dysregulation and elevated GCs, these results suggest that stress‐related signalling may indirectly accelerate oligodendrocyte maturation in human cortical circuits.

Taken together, these studies illustrate how GCs and ELS can influence neural lineage outcomes through indirect routes, ranging from progenitor subtype skews and selective vulnerability of neuronal subpopulations to shifts in maturation within lineages. Unlike the GC‐linked fate bias described earlier, these examples highlight more nuanced alterations in proliferation dynamics, transcriptional states, and within‐lineage balance that cumulatively shape brain architecture. While the broader consequences of stress and GC signalling for neurogenesis, gliogenesis and circuit remodelling have been investigated across many systems and species, these exemplar studies are focused more directly to fate‐related processes, linking stress physiology to enduring changes in brain cell composition.

## 5 UNDERLYING MECHANISMS OF GLUCOCORTICOID EFFECTS ON CELL FATE

ELS and GCs may influence neural cell fate, ranging from direct alterations in progenitor decisions to more indirect effects mediated through alterations to neurogenesis dynamics. A central challenge is to delineate the molecular and cellular mechanisms by which GC exposure produces these outcomes. GCs act through a rich set of signalling modes that extend well beyond simple receptor–DNA interactions, intersecting with transcriptional, epigenetic, metabolic and inflammatory processes that collectively shape the trajectories of neural progenitors. Moreover, many of these mechanisms operate at multiple levels, from intrinsic programming within progenitor cells to modulation of local niche signals and systemic influences.

The capacity of GCs to shape neural development is largely mediated through their intracellular receptors, the GR and MR. Both receptors are ligand‐activated transcription factors, yet differ in their affinity for GCs and in the transcriptional programmes they engage. MR, with its high affinity, is typically occupied even under basal GC concentrations and has been implicated in maintaining neuronal excitability and basal stress responsivity.^9^ In vitro, MR is known to mediate neuronal differentiation and have neuroprotective effects since MR overexpression in embryonic stem cell‐derived neurons promotes neuronal differentiation, increases anti‐apoptotic signalling, and reduces cell death.^74^ Meanwhile, in the hippocampal CA2 region, MR is essential for maintaining the molecular identity of pyramidal neurons, where conditional MR knockout leads to loss of CA2‐specific markers, altered synaptic properties, and disrupted behaviours linked to CA2 function.^75^

GR, by contrast, is recruited under conditions of stress when circulating GC levels are elevated, and its broader distribution of genomic and non‐genomic actions makes it a key mediator of stress‐induced reprogramming.^76^ Activation of GR, especially by synthetic GCs, alters gene expression in neurons and progenitor cells, affecting differentiation and maturation pathways.^77^ Meanwhile, in the pancreas, GR activation in vitro plays a significant role in early‐stage progenitor cell differentiation in mice and humans, promoting specific fate trajectories and influencing lineage commitment.^78^ These studies suggest that GR and MR signalling may also mediate cell fate in the brain, integrating environmental signals to regulate neuronal development, plasticity, and stress responses in a context‐ and cell‐type‐specific manner.

Beyond receptor activation, GCs can exert longer‐lasting influences on cell fate through epigenetic regulation. By modifying the epigenetic landscape through alteration of chromatin accessibility, DNA methylation, histone modifications, and gene expression programmes, GCs have the potential to modify developmental trajectories long after the initial hormonal signal has dissipated. For example, specific epigenetic modifications, such as DNA methylation and histone modifications, regulate the proliferation of cortical progenitors and the timing of their differentiation into various neuronal subtypes.^79^ These epigenetic changes control dynamic patterns of gene expression during cortical development, ensuring that progenitor cells switch fate at the appropriate developmental stage and location. By orchestrating when and where genes are turned on or off, such epigenetic mechanisms provide a flexible and reversible means to guide neural progenitor cells towards specific lineages, ultimately shaping the diversity and function of neurons in the cortex.

Berry et al.^80^ demonstrated that prenatal exposure to GCs reprogrammes NSPCs in the developing brain. Using genome‐wide assays, they found that GR binds preferentially to specific regions of accessible chromatin, influencing gene expression programmes that are critical for cell fate decisions. The study also identifies SOX2 as a transcription factor that modulates the genomic response of certain GR target genes to GCs, further linking transcriptional regulation and chromatin accessibility to the determination of neural cell fate. As such, disruptions in these epigenetic processes can lead to abnormal cell fate decisions, which may contribute to neurodevelopmental disorders.

Epigenetic modifications do not operate in isolation but instead converge with signalling pathways that are themselves central regulators of cell fate. A growing body of research supports that GCs can directly modulate neurotrophin signalling in the brain, including BDNF (brain‐derived neurotrophic factor),^32^ a critical regulator of neuronal differentiation and survival.^81^ GCs and BDNF interact at multiple levels: GCs can regulate BDNF expression, influence its intracellular transport and degradation, and share common downstream signalling pathways, affecting neurodevelopment, synaptic plasticity, and cellular homeostasis.^82^ For example, chronic GC exposure in a cortico‐hippocampal network‐on‐a‐chip system slows the transport of BDNF in cortical axons, which is necessary for BDNF delivery to the hippocampus, leading to reduced levels of BDNF.^83^ The authors link this with decreased proliferation, survival, and maturation of new neurons under chronic GC exposure, via a mechanism involving phosphorylation of huntingtin (HTT) protein, which impairs BDNF vesicle movement. These findings highlight how GC–BDNF interactions can disrupt the trophic support required for neuronal differentiation and maturation, thereby influencing the overall capacity of neurogenesis under stress conditions.

Taken together, these studies highlight that GC effects on brain cell fate do not arise from a single mechanism but from the convergence of receptor‐mediated transcriptional programmes, epigenetic remodelling, and cross‐talk with developmental and signalling pathways. The resulting outcomes are highly context‐dependent, varying with developmental stage, brain region, and cell type. A key unresolved issue is how transient hormonal signals become embedded into long‐term developmental trajectories, altering the balance of intrinsic programmes and extrinsic cues that guide progenitor behaviour.

## 6 FUTURE PERSPECTIVES AND CHALLENGES

Looking ahead, a central challenge is to define precisely how GCs might reshape lineage allocation in vivo and to disentangle direct fate influences from broader effects on proliferation, survival, and maturation. Evidence from human organoids, rodent hippocampus, and zebrafish hypothalamus suggests that GCs can bias progenitor trajectories, shift subtype balance, and alter maturation pace. Yet the precise nature, timing, and persistence of these fate‐shaping influences remain unclear. Progress will require integrating mechanistic insights with new tools, such as single‐cell approaches, advanced lineage tracing, and cross‐species comparisons, to understand how stress hormones embed experience into the cellular architecture of the brain.

Recent advances in single‐cell and spatial technologies have the capacity to transform the study of how external stimuli influence cell fate in the brain. Single‐cell RNA sequencing (scRNA‐seq) enables high‐throughput profiling of transcriptional states in thousands of progenitors and their progeny, reconstructing developmental trajectories and fate transitions, though at the cost of spatial context.^84^ Meanwhile, complementary approaches such as single‐nucleus epigenomic profiling, including DNA methylation and chromatin accessibility assays, can provide insight into how experience reshapes the regulatory landscape underlying fate specification.^85^ Spatial transcriptomics adds a further layer by retaining positional information within intact tissue, enabling the mapping of cell fate decisions within the architectural context of the developing brain.^84^ Alongside these population‐scale methods, lineage‐tracing approaches such as Mosaic Analysis with Double Markers (MADM)^86^ and ProTracer^87^ now permit in vivo tracking of proliferative history and fate outcomes of specific progenitor cohorts in the intact brain, allowing effects on lineage allocation to be disentangled from secondary survival or proliferation effects. Together, these approaches enable a multi‐resolution view of cell fate, from molecular programming to spatial positioning, bringing the field closer to defining how transient hormonal cues are translated into enduring developmental trajectories.

In parallel, computational modelling provides a complementary approach for probing how environmental stimuli such as GCs bias lineage trajectories. Mathematical models can integrate diverse biological inputs, from transcriptional programmes to extracellular signalling, to predict how progenitors resolve fate decisions.^88^, ^89^ Landscape models, inspired by Waddington's epigenetic landscape, abstract complex gene‐regulatory networks into more tractable ‘fate valleys’ enabling simulation of how transient signalling pulses might tilt developmental trajectories towards alternative outcomes.^90^ Such models not only generate experimentally testable predictions, including non‐intuitive effects of signal timing or strength, but also provide a framework for integrating datasets from scRNA‐seq, epigenomics, and lineage tracing into coherent fate maps. By bridging mechanistic experiments with predictive theory, these approaches can help to reveal how stress hormones interact with intrinsic developmental programmes to shape brain cell fate in ways that may be difficult to observe directly.

Recent lineage tracing work has provided seminal insights into the temporal dynamics of cortical progenitor behaviour, showing that radial glial cells follow an intrinsic programme in which early‐born deep‐layer neurons are generated first, followed by upper‐layer neurons, and eventually a switch from neurogenesis to gliogenesis.^91^, ^92^ These findings highlight that progenitor fate is not only determined by identity but also by precise timing, with disruptions to these schedules likely able to produce marked changes in cortical architecture. It is plausible that ELS and GC exposure could perturb these temporal programmes, for example by accelerating or delaying the onset of gliogenesis, or by altering numbers of neurons contributing to different cortical layers, which could ultimately contribute to structural alterations such as the cortical thinning observed in GC‐exposed children.^23^ Whether similar principles could extend to other brain regions remains largely unexplored. However, in the hippocampus it is known that during embryogenesis the temporal origins of neurons contribute to anatomical and functional diversity^93^, ^94^ and hippocampal neurogenesis is also highly sensitive to GC signalling.^8^, ^33^ Despite this, studies with the single‐cell resolution or advanced fate‐tracing tools have yet to be applied to the study of GC‐induced alteration of hippocampal neurogenesis. Application of advanced single cell profiling and lineage analysis methods to experimental systems where GC exposure can be precisely manipulated has the potential to reveal unprecedented insights into how stressful experiences contribute to altered brain structure and function.

Disrupted fate dynamics under conditions of ELS have important implications for psychiatric and neurological disorders. Aberrant allocation of excitatory versus inhibitory neurons could contribute to the altered network balance reported in depression and schizophrenia,^95^, ^96^ while potential shifts in glial output in psychiatric disease^97^, ^98^ may affect synaptic pruning, myelination, and neuroinflammation. Human imaging studies consistently link childhood adversity with cortical thinning^99^ and hippocampal volume loss,^100^ which could in part reflect cumulative alterations in fate timing and lineage allocation as discussed above. These insights raise the possibility of novel interventions to prevent or mitigate associated neuropsychiatric symptoms, such as targeting GR/MR signalling to prevent reprogramming, modulating epigenetic regulators to restore progenitor competence, or enhancing trophic signalling through BDNF pathways to stabilise neuronal differentiation. Translating these mechanistic insights into therapeutic strategies will require bridging controlled experimental models with human longitudinal data, but the conceptual framework of cell fate disruption provides a new lens through which to think about the long‐term consequences of ELS exposure.

Despite rapid progress, many questions remain unresolved. We still lack a clear understanding of how the dose, duration, and developmental timing of GC exposure interact to shape progenitor fate, or whether these changes are reversible once developmental windows have closed. Sex differences, which are well‐documented in stress‐related disorders,^101^ are also likely to influence fate trajectories, yet remain understudied at the cellular level. Moreover, translating insights from animal and organoid models to the human brain will require careful attention to species‐specific developmental programmes. Ultimately, the intersection of stress biology and developmental neuroscience represents a frontier in which cellular fate decisions become a nexus linking experience to brain architecture and lifelong mental health.

## CONFLICT OF INTEREST STATEMENT

The author declares no conflicts of interest.

## Data Availability

Data sharing not applicable to this article as no datasets were generated or analysed during the current study.
